# Maximum Entropy on the Complex Probability Framework

**DOI:** 10.3390/e28101055

**Published:** 2026-09-25

**Authors:** Francisco J. Tapiador

**Affiliations:** Institute of Environmental Sciences (ICAM), University of Castilla–La Mancha (UCLM), Avda. Carlos III s/n, 45071 Toledo, Spain; francisco.tapiador@uclm.es

**Keywords:** complex probability, maximum entropy, Jaynes, Gibbs distribution, complex Lagrange multipliers, Kolmogorov axioms, 60A05, 94A17

## Abstract

Jaynes’ maximum entropy (MaxEnt) principle selects, from all probability distributions consistent with a set of constraints, the one that maximizes the Shannon entropy. The principle is well-defined for classical, real-valued probabilities, but extending it to complex-valued probability theories requires care. Youssef’s complex probability framework, in which quantum mechanics is reformulated as a Bayesian theory with complex amplitudes and the observable probability is the squared modulus (Born rule), does not admit a natural MaxEnt extension: the $L^{2}$ normalization of that framework is incompatible with the $L^{1}$ normalization assumed by Shannon entropy, and the resulting complex-valued entropy functional cannot be maximized without additional structure absent from Youssef’s axioms. The present paper proposes a different extension of Kolmogorov’s axioms to the complex domain, replacing non-negativity with the condition that the real part of every probability be non-negative. This framework, which retains $L^{1}$ normalization and recovers classical probability when the imaginary part vanishes, admits a natural MaxEnt principle: the real part of the complex Shannon entropy is a well-posed real functional, its Euler–Lagrange equations produce an explicit complex Gibbs distribution with complex Lagrange multipliers, and the classical Gibbs distribution is recovered as the special case of real multipliers. The die problem (Jaynes’ Brandeis dice example) is worked out in detail for both the fair and the loaded cases, illustrating how imaginary constraints redistribute the observable probability mass in a way that has no counterpart in classical MaxEnt.

## 1. Introduction

### 1.1. Classical Maximum Entropy

The principle of maximum entropy, introduced by Jaynes [1,2] and grounded in Shannon’s information theory [3], provides a principled method for selecting a probability distribution from among all those consistent with a set of constraints. The selected distribution maximizes the Shannon entropy

H[p]=−∫p(x)logp(x)dx,

subject to normalization ∫p(x)dx=1 and moment constraints $\int g_{k}\left(x\right)\;p\left(x\right)\;dx=c_{k}$. The solution is the exponential family, or Gibbs distribution, $p^{*}\left(x\right)\propto \exp\left(-\sum_{k}\lambda_{k}g_{k}\left(x\right)\right)$, where the $\lambda_{k}\in R$ are Lagrange multipliers determined by the constraints. This framework, together with its Bayesian interpretation [4], is the standard approach to inference under incomplete information.

The scope of MaxEnt in physics has broadened substantially since Jaynes’ original formulation. Shore and Johnson [5] placed the principle on an axiomatic foundation by showing that it is the unique updating procedure consistent with a set of consistency axioms, removing its dependence on the particular motivation of entropy as a measure of uncertainty. The review of Pressé et al. [6] surveys the range of applications across equilibrium and non-equilibrium statistical physics, from standard Boltzmann–Gibbs thermodynamics through to maximum caliber, the path-entropy counterpart of MaxEnt for dynamical trajectories. Quantum applications continue to be studied (e.g., [7]).

Among specific applications, MaxEnt has been used to derive fluctuation relations in non-equilibrium systems, to reconstruct spectral functions from imaginary-time quantum Monte Carlo data, and to infer interaction networks in complex systems from marginal distribution data. Recent work illustrates both directions: Caticha and Golan [8] developed an entropic inference framework for economic modeling using MaxEnt as the governing principle, with Lagrange multipliers emerging as prices in a statistical equilibrium, and Ghavasieh and De Domenico [9] proposed a maximum entropy principle for network states that characterizes systems with heterogeneous, correlated connectivity patterns, using von Neumann entropy of network density matrices as the objective functional. The method has also been applied in the environmental sciences [10,11]. Indeed, new MaxEnt methods and refinements continue to appear; representative recent examples include [12,13,14,15].

The present paper develops the MaxEnt principle for complex probabilities independently and in full, deriving the complex Gibbs distribution with complex Lagrange multipliers from first principles, connecting it to the axiomatic structure of the complex probability framework, and illustrating its consequences through the die problem (the infamous Jaynes’ Brandeis dice example; note that the singular “die” is used throughout).

### 1.2. Prior Approaches to Complex Probability

Extending probability to complex-valued functions has been proposed in several directions, which differ sharply in their relationship to MaxEnt.

Youssef [16,17] proposes retaining Cox’s Bayesian axioms [18] for probability, extending the range of the probability function from [0,1] to C. No non-negativity condition is imposed on the complex probabilities themselves; the observable, classical probability of an event *A* is recovered as $\mathrm{Prob}\left(A\right)={\left|P\left(A\right)\right|}^{2}$, the Born rule. Normalization in Youssef’s framework is therefore $\sum_{k}{\left|P\left(k\right)\right|}^{2}=1$, an $L^{2}$ condition. Youssef shows that this framework recovers the Schrödinger equation, the superposition principle, and the path-integral representation of quantum mechanics from probabilistic axioms alone.

Rodriguez [19] generalizes Youssef’s program by replacing C with an arbitrary finite-dimensional Clifford algebra *G*. A Clifford-algebra-valued conditional measure ψ:A→G is introduced, and it is shown that the resulting spaces of such measures are Hilbert spaces. The real numbers, complex numbers, quaternions, and spinors all appear as special cases of the Clifford structure, so the framework subsumes both classical probability (real-valued) and quantum mechanics (complex-valued) in a single algebraic setting. Rodriguez derives the Born rule and the Schrödinger equation from the Markov property, demonstrating that the Clifford extension retains the essential properties of Youssef’s framework while broadening its algebraic scope. The paper was presented at the MaxEnt 1998 workshop in Garching, showing awareness of the Bayesian MaxEnt community, yet the framework does not develop a MaxEnt principle within Clifford-valued probability for the same structural reasons that prevent MaxEnt from extending to Youssef’s complex-valued version.

Indeed, three obstacles arise when one attempts to apply MaxEnt within either Youssef’s or Rodriguez’s framework.

First, the normalization condition is $\sum_{k}{\left|P\left(k\right)\right|}^{2}=1$, an $L^{2}$ condition in both Youssef’s and Rodriguez’s frameworks, whereas the Shannon entropy is naturally adapted to an $L^{1}$ normalization $\sum_{k}P\left(k\right)=1$. A probability distribution $p_{k}={\left|P\left(k\right)\right|}^{2}$ satisfying $L^{1}$ normalization and a complex amplitude P(k) satisfying $L^{2}$ normalization are different objects. Maximizing Shannon entropy over {P(k)} subject to $\sum |P_{k}{|}^{2}=1$ amounts to maximizing it over the observable Born probabilities $p_{k}={\left|P\left(k\right)\right|}^{2}$, leaving the phases of P(k) entirely unconstrained. The imaginary part of the complex probability plays no role in the entropy and carries no MaxEnt content.

Second, if one instead defines a complex entropy functional $H=-\sum_{k}P\left(k\right)\log P\left(k\right)\in C$ and attempts to maximize it, the result is complex-valued, and C carries no total order. Maximizing a complex-valued functional requires specifying what “maximum” means in C, a specification absent from Youssef’s axioms.

Third, the constraints in Youssef’s framework are naturally stated as expectations of the Born probabilities, $\sum_{k}{\left|P\left(k\right)\right|}^{2}g_{k}=c_{k}$, which are real. But the MaxEnt solution would then be the classical Gibbs distribution for $p_{k}={\left|P\left(k\right)\right|}^{2}$, with the phases of P(k) freely choosable. Maximizing entropy over the phases would require a separate principle, which Youssef does not provide. The last sentence of Youssef’s 1994 paper acknowledges this gap explicitly, noting that a suitable maximum entropy principle has already been proposed and successfully used in many-body problems, with a citation to nuclear physics work by Canosa, Rossignoli and Plastino [20,21]. Those papers apply the von Neumann entropy S=−Tr(ρlogρ) to density matrices ρ satisfying ρ≥0 and Tr(ρ)=1, a formalism entirely within standard quantum mechanics and unrelated to Youssef’s complex probability amplitudes. The MaxEnt principle Youssef acknowledges is one that operates on density matrices with the Born-rule normalization and the positive-semidefiniteness condition, not on the complex-valued probabilities his axioms define. No MaxEnt principle is derived from those axioms in his paper.

### 1.3. The Present Framework

The Kolmogorov–Jaynes program of extending MaxEnt while preserving the measure-theoretic foundations has an earlier precedent in [22], which applied Jaynes’ MaxEnt principle in an applied remote sensing setting. That doctoral dissertation established MaxEnt as a practical inference tool for satellite image data fusion but did not develop the complex-valued probability framework there. The present paper provides the fully fledged formalism and derives consequences beyond the original realm.

The key to the formalism is the replacement of Kolmogorov’s non-negativity axiom P(A)≥0 with the weaker condition Re(P(A))≥0, retaining $L^{1}$ normalization P(Ω)=1. The change is not trivial. The observable classical probability of *A* is Re(P(A)), recovered by a linear operation. This has three consequences that are relevant for MaxEnt.

First, the normalization remains $L^{1}$, so Shannon entropy is immediately applicable to the complex density.

Second, the entropy H[f]=−∫flogfdx is complex-valued, but its real part Re(H[f]) is a well-defined real functional that reduces to the classical Shannon entropy when Im(f)=0. The MaxEnt problem becomes: maximize Re(H[f]) subject to the constraints.

Third, the Euler–Lagrange equations for this real functional produce an explicit solution: the complex Gibbs distribution with complex Lagrange multipliers $\Lambda_{k}\in C$, whose real parts reproduce the classical Gibbs distribution when the imaginary parts vanish.

The rest of the paper develops the formalism that extends MaxEnt to the complex domain. Section 2 states the axioms and their immediate consequences. Section 3 derives the MaxEnt principle and the complex Gibbs distribution. Section 4 works out the classical die example in detail. Section 5 summarizes and states open questions.

## 2. The Complex Probability Framework

### 2.1. Axioms

Let (Ω,F) be a measurable space. The classical Kolmogorov probability measure [23] P:F→R satisfies non-negativity P(A)≥0, normalization P(Ω)=1, and countable additivity. The present framework retains normalization and countable additivity but replaces non-negativity with a condition compatible with the field structure of C.

**Definition** **1** (Complex probability measure)**.** *A complex probability measure on (Ω,F) is a function P:F→C satisfying:*

*(CP1)*  *Re(P(A))≥0 for all A∈F;*
*(CP2)*  *P(Ω)=1;*
*(CP3)*  *For pairwise disjoint ${\left\{A_{i}\right\}}_{i=1}^{\infty }\subset F$, $P\;\left({⋃}_{i}A_{i}\right)=\sum_{i}P\left(A_{i}\right)$, convergence in C.*

Condition (CP1) is the unique extension of non-negativity that is meaningful in C, for C admits no total order compatible with its field structure. The classical axioms are recovered by setting Im(P)≡0.

**Proposition** **1** (Structural consequences)**.** *Let P be a complex probability measure. For any A∈F:*

*1.*  *P(Ø)=0;*
*2.*  *$P\left(A^{c}\right)=1-P\left(A\right)$, so Re(P(A))∈[0,1] and Im(P(Ac))=−Im(P(A));*
*3.*  *The total imaginary mass vanishes: Im(P(Ω))=0.*

**Proof.**  All three items follow from (CP2), (CP3), and the decomposition $\Omega =A\cup A^{c}$.    □

### 2.2. Complex Probability Density Functions

**Definition** **2** (Complex PDF)**.** *A function f:R→C is a complex probability density function (CPDF) if:*

*(D1)*  *Re(f(x))≥0 for all x;*
*(D2)*  *$\int_{-\infty }^{\infty }\left|f\left(x\right)\right|\;dx<\infty$;*
*(D3)*  *$\int_{-\infty }^{\infty }f\left(x\right)\;dx=1$, i.e., ∫Re(f(x))dx=1 and ∫Im(f(x))dx=0.*

*Writing f=u+iv, conditions (D1)–(D3) require u≥0, ∫u(x)dx=1, and ∫v(x)dx=0: the real part u is a classical PDF, and the imaginary part v is a zero-mean signed density.*

**Remark** **1** (Relation to Youssef’s and Rodriguez’s frameworks)**.** *The normalization ∫f(x)dx=1 here differs fundamentally from Youssef’s Born-rule normalization ${\int |f\left(x\right)|}^{2}\;dx=1$ and from Rodriguez’s Clifford-algebra norm. In the present framework, Re(P(A))=∫Audx≥0 is the observable classical probability. In Youssef’s and Rodriguez’s frameworks, the observable probability is $\int_{A}{\left|f\right|}^{2}\;dx$. These are different operations on f, and they lead to different entropy functionals, as the introduction establishes.*

### 2.3. The Operational Meaning of the Imaginary Component

Because *u* is a classical density and *v* is a signed density of zero total mass, one can ask what *v* represents. There are three readings.

*A latent binary partition.* Since ∫vdx=0, the Hahn–Jordan theorem decomposes *v* uniquely as $v=q^{\left(+\right)}-q^{\left(-\right)}$ into non-negative parts of equal total mass $m=\int q^{\left(\pm \right)}\;dx$. Writing $\pi^{\left(\pm \right)}=q^{\left(\pm \right)}/m$ for the normalized parts, the imaginary moment becomes

(1) $$c^{\left(v\right)}=\int g\;v\;dx=m\left(E_{\pi^{\left(+\right)}}\left[g\right]-E_{\pi^{\left(-\right)}}\left[g\right]\right),$$

that is, the contrast in expected constraint value between two latent sub-populations carrying equal prior weight. Prescribing an imaginary moment is therefore prescribing how far apart two unobserved sub-populations lie.

*A direction orthogonal to all frequency measurement.* Any classical observation is a functional of the empirical frequencies and hence of *u* alone. The imaginary part lies in the set {v:∫vdx=0}, the orthogonal complement of the constant function, so no sequence of observations of the experiment can determine $c^{\left(v\right)}$. It is prior structural information, not an estimable parameter, and the framework should be read as providing a place to record such information rather than as extracting it from data.

*A departure from detailed balance.* For a stationary Markov chain on the support with marginal *u*, the probability current across the bond between adjacent states is proportional to the local average of *v*. The current vanishes at detailed balance, so $c^{\left(v\right)}=0$ characterizes an equilibrium prior and $c^{\left(v\right)}\neq 0$ a non-equilibrium one. This reading gives the sign of $c^{\left(v\right)}$ a direction in state space, which is the property exploited in Section 4.

The three readings are projections of one object rather than alternatives, and they agree numerically the examples computed below.

### 2.4. Comparison of Frameworks and Non-Triviality of the Extension

The present framework occupies a position between classical Kolmogorov probability (the special case Im(f)≡0) and Youssef’s framework (which imposes no non-negativity condition at all). Table 1 summarises the three frameworks side by side.

The modification from P(A)≥0 to Re(P(A))≥0 may appear minor, but its mathematical and inferential consequences are substantial, as follows.

#### 2.4.1. The Sample Space of Probability Values Changes Topology

Classical probability takes values in the compact interval [0,1]. Complex probability takes values in the closed right half-plane {z∈C:Re(z)≥0}, which is unbounded. The characteristic function of a classical probability measure is positive definite (Bochner’s theorem), a statement that fails for complex probability measures, as the Fourier transform of the imaginary component is a signed measure. The entire theory of characteristic functions must therefore be rebuilt for the complex case, with Bochner’s theorem replaced by a pair of separate conditions on the real and imaginary Fourier components.

#### 2.4.2. Derived Quantities Need Not Satisfy (CP1)

Although (CP1) is an axiom for the measure itself, it is not inherited automatically by derived objects. Conditional complex probabilities P(A|B)=P(A∩B)/P(B) can have negative real part even when *P* satisfies (CP1), as the following argument shows. The classical conditional probability P(A|B)=P(A∩B)/P(B) is always non-negative when P(B)>0, since P(A∩B)≤P(B). For complex *P*, the division P(A∩B)/P(B) involves complex arithmetic. Writing P(A∩B)=α+iβ and P(B)=γ+iδ with α,γ≥0, the real part of the quotient is $\left(\alpha \gamma +\beta \delta \right)/\left(\gamma^{2}+\delta^{2}\right)$, which can be negative when βδ<0 and |βδ|>αγ. Independence and conditional independence therefore require more care in the complex framework than in the classical one.

#### 2.4.3. The Moment Problem Splits

In classical probability, the Hamburger moment problem asks: given a sequence of real numbers ${\left\{\mu_{n}\right\}}_{n\in N}$, does a non-negative measure with those moments exist? The answer is governed by positive semidefiniteness of the Hankel matrix. For complex probability, the moment problem decomposes into a classical sub-problem for the real moments {Re(μn)}n∈N (with the same Hankel condition) and a signed-measure sub-problem for the imaginary moments {Im(μn)}n∈N, for which no positivity condition applies. The imaginary moments can therefore take arbitrary values subject only to the zero-total-mass condition Im(μ0)=0. This decoupling means that the imaginary part of the distribution carries genuinely new information, unconstrained by the classical moment conditions that govern the real part.

#### 2.4.4. The MaxEnt Solution Requires a New Admissibility Condition

For classical MaxEnt, the Gibbs distribution $p^{*}\propto e^{-\sum \lambda_{k}g_{k}}$ is always non-negative for real $\lambda_{k}$, so no additional condition is needed. For complex MaxEnt, the complex Gibbs distribution $f^{*}\propto e^{-\sum \Lambda_{k}g_{k}}$ with $\Lambda_{k}\in C$ is not automatically a valid CPDF: condition (D1) imposes a geometric constraint on the imaginary multipliers βk=−Im(Λk). This constraint, which we call the admissibility condition, is the central new ingredient of complex MaxEnt and has no counterpart in the classical theory. Its content and consequences are developed in detail in Section 3.7.

## 3. Maximum Entropy with Complex Probabilities

This section formalizes the maximum entropy method with complex probabilities. Let us first recall the classical MaxEnt: for a PDF *p* on R with p≥0 and ∫p(x)dx=1, Jaynes’ MaxEnt problem with *K* constraint functions $g_{1},\ldots ,g_{K}$ and prescribed moments $c_{1},\ldots ,c_{K}\in R$ is

(2) $$\max_{p}\;H\left[p\right]=-\int p\left(x\right)\log p\left(x\right)\;dx\;\mathrm{subject}\;\mathrm{to}\;\int g_{k}\left(x\right)\;p\left(x\right)\;dx=c_{k},\;k=1,\ldots ,K.$$

The solution, obtained by Lagrange multipliers $\lambda_{0},\alpha_{1},\ldots ,\alpha_{K}\in R$, is

(3) $$p^{*}\left(x\right)=\frac{1}{Z(\alpha )}\exp\;\left(-\sum_{k=1}^{K}\alpha_{k}g_{k}\left(x\right)\right),\;Z\left(\alpha \right)=\int \exp\;\left(-\sum_{k=1}^{K}\alpha_{k}g_{k}\left(x\right)\right)dx,$$

with $-\partial \log Z/\partial \alpha_{k}=c_{k}$.

### 3.1. The Complex Entropy Functional

The new framework is as follows. For a CPDF f=u+iv, we extend the Shannon entropy to

(4) $$H\left[f\right]:=-\int_{-\infty }^{\infty }f\left(x\right)\log f\left(x\right)\;dx\;\in \;C,$$

where log denotes the principal branch of the complex logarithm. Writing $f=|f|\;e^{i\theta }$ with $\left|f\right|=\sqrt{u^{2}+v^{2}}$ and θ=arctan(v/u), the real part of H[f] is

(5) Re(H[f])=−∫u(x)log|f(x)|−v(x)θ(x)dx.

When Im(f)≡0, this reduces to −∫ulogudx, the classical Shannon entropy. The imaginary part *v* contributes through the phase θ: a non-trivial imaginary part can increase the real entropy relative to the purely real case.

Since C carries no total order, maximizing H[f] directly is ill-posed. The natural resolution is to maximize Re(H[f]), which is a well-defined real functional, reduces to Shannon entropy in the classical limit, and is the only component of H[f] accessible to observation, since observable probability is Re(P).

### 3.2. The Complex MaxEnt Problem

Given constraint functions $g_{1},\ldots ,g_{K}$ and prescribed complex moments $c_{k}=c_{k}^{\left(u\right)}+ic_{k}^{\left(v\right)}\in C$, the complex MaxEnt problem is

(6) maxf=u+ivRe(f)≥0,∫fdx=1Re(H[f])subjectto∫gk(x)f(x)dx=ck,k=1,…,K.

The constraint $\int g_{k}f\;dx=c_{k}$ decomposes into a real part $\int g_{k}u\;dx=c_{k}^{\left(u\right)}$ and an imaginary part $\int g_{k}v\;dx=c_{k}^{\left(v\right)}$. Treating *u* and *v* as independent real functions, the Lagrangian is

(7) $$\begin{array}{cc} L[u,v] & =-\int \left(u\log|f|-v\theta \right)dx-\lambda_{0}\;\left(\int u\;dx-1\right)-\mu_{0}\int v\;dx\\ & \;-\sum_{k=1}^{K}\;\left[\alpha_{k}\;\left(\int g_{k}u\;dx-c_{k}^{\left(u\right)}\right)+\beta_{k}\;\left(\int g_{k}v\;dx-c_{k}^{\left(v\right)}\right)\right], \end{array}$$

with real multipliers $\lambda_{0},\mu_{0},\alpha_{1},\ldots ,\alpha_{K},\beta_{1},\ldots ,\beta_{K}\in R$.

### 3.3. Functional Derivatives

The Lagrangian (7) is stationary when its variations with respect to *u* and *v* vanish independently. Obtaining those variations requires the functional derivatives of Re(H[f]), which are derived here in full because the complex case differs from the classical one in two respects: the entropy density is a function of two real fields rather than one, and the phase contributes a term that has no classical counterpart.

#### 3.3.1. The Real Entropy Density

Write f=u+iv in polar form, $f=\left|f\right|e^{i\theta }$, with 

(8) $$\left|f\right|=\sqrt{u^{2}+v^{2}},\;\theta =\arg\left(f\right)=\arctan\;\left(\frac{v}{u}\right).$$

On the principal branch, logf=log|f|+iθ, so

(9) $$f\log f=\left(u+iv\right)\left(\log|f|+i\theta \right)=\left(u\log|f|-v\theta \right)+i\left(u\theta +v\log|f|\right),$$

and taking real parts gives the entropy density

(10) Φ(u,v):=Reflogf=ulog|f|−vθ=u2logu2+v2−varctanvu,

so that Re(H[f])=−∫Φ(u,v)dx, which is Equation (5).

Two conditions delimit the domain of validity. The principal branch requires f∉(−∞,0], which (CP1) guarantees away from f=0 since u≥0 confines θ to [−π/2,π/2]. The representation of θ by arctan(v/u) additionally requires u>0, so the derivation below holds pointwise wherever the observable density is strictly positive. The set {u=0} is precisely the boundary at which the admissibility condition becomes active, and it is treated in Section 3.7 rather than here.

#### 3.3.2. Reduction to Partial Derivatives

The density Φ depends on *u* and *v* pointwise and contains no derivatives of either field. The Euler–Lagrange operator therefore reduces to a partial derivative,

(11) $$\frac{\delta }{\delta u}\int \Phi \left(u,v\right)\;dx=\frac{\partial \Phi }{\partial u},\;\frac{\delta }{\delta v}\int \Phi \left(u,v\right)\;dx=\frac{\partial \Phi }{\partial v},$$

and consequently δRe(H)/δu=−∂Φ/∂u and δRe(H)/δv=−∂Φ/∂v. The constraint terms in (7) are linear in *u* and *v*, so their variations are $g_{k}$ and 1 multiplied by the corresponding multipliers, which is why the whole difficulty resides in Φ.

It is worth noting why *u* and *v* must be varied separately instead of differentiating with respect to *f* directly. The functional Re(H[f]) is real-valued and therefore not holomorphic in *f*, so no complex derivative dH/df exists in the ordinary sense. The pair (u,v) supplies the two independent real coordinates that the variational problem requires.

#### 3.3.3. Derivative with Respect to the Real Part

Differentiate the two terms of (10) in turn. For the first, apply the product rule together with $\partial_{u}\log\sqrt{u^{2}+v^{2}}=u/\left(u^{2}+v^{2}\right)$:

(12) $$\frac{\partial }{\partial u}\left(u\log\sqrt{u^{2}+v^{2}}\right)=\log\sqrt{u^{2}+v^{2}}+u\cdot \frac{u}{u^{2}+v^{2}}=\log\left|f\right|+\frac{u^{2}}{u^{2}+v^{2}}.$$

For the second, note that *v* is held fixed and that

(13) $$\frac{\partial }{\partial u}\arctan\;\left(\frac{v}{u}\right)=\frac{1}{1+v^{2}/u^{2}}\cdot \left(-\frac{v}{u^{2}}\right)=-\frac{v}{u^{2}+v^{2}},$$

whence

(14) $$\frac{\partial }{\partial u}\left(-v\arctan\frac{v}{u}\right)=-v\cdot \left(-\frac{v}{u^{2}+v^{2}}\right)=\frac{v^{2}}{u^{2}+v^{2}}.$$

Adding (12) and (14), the two fractions combine into unity because their numerators sum to the common denominator:

(15) $$\frac{\partial \Phi }{\partial u}=\log|f|+\frac{u^{2}+v^{2}}{u^{2}+v^{2}}=\log\left|f\right|+1.$$

The functional derivative of the entropy is the negative of this quantity, which establishes

(16) δRe(H)δu=−log|f|+1.

#### 3.3.4. Derivative with Respect to the Imaginary Part

Differentiating the first term of (10) with respect to *v*, now with *u* held fixed, gives

(17) $$\frac{\partial }{\partial v}\left(u\log\sqrt{u^{2}+v^{2}}\right)=u\cdot \frac{v}{u^{2}+v^{2}}=\frac{uv}{u^{2}+v^{2}}.$$

The second term requires the product rule, since both factors depend on *v*. Using

(18) $$\frac{\partial }{\partial v}\arctan\;\left(\frac{v}{u}\right)=\frac{1}{1+v^{2}/u^{2}}\cdot \frac{1}{u}=\frac{u}{u^{2}+v^{2}},$$

one obtains

(19) $$\frac{\partial }{\partial v}\left(-v\arctan\frac{v}{u}\right)=-\arctan\frac{v}{u}-v\cdot \frac{u}{u^{2}+v^{2}}=-\theta -\frac{uv}{u^{2}+v^{2}}.$$

The rational terms of (17) and (19) cancel exactly, leaving only the phase:

(20) $$\frac{\partial \Phi }{\partial v}=\frac{uv}{u^{2}+v^{2}}-\theta -\frac{uv}{u^{2}+v^{2}}=-\theta .$$

Changing sign gives the second functional derivative,

(21) δRe(H)δv=θ=arg(f).

The cancellation in (20) is the structural reason why the stationarity condition for the phase is algebraically simple. Whatever the amplitude of *f*, the variation of the real entropy with respect to the imaginary part is the phase itself, so setting δL/δv=0 produces a phase that is linear in the constraint functions.

#### 3.3.5. Consistency Checks

Three checks confirm (16) and (21).

First, the classical limit. Setting v≡0 gives |f|=u and θ=0, so (16) becomes −(logu+1), the familiar variation of the Shannon entropy that yields the classical Gibbs distribution, while (21) vanishes identically. The imaginary channel therefore contributes nothing when it is empty, as it must.

Second, the two real derivatives reassemble into a single complex expression. Combining (15) and (20),

(22) $$\frac{\partial \Phi }{\partial u}-i\;\frac{\partial \Phi }{\partial v}=\left(\log|f|+1\right)+i\theta =\log f+1,$$

so that

(23) δRe(H)δu−iδRe(H)δv=−1+logf.

The right-hand side of (23) is the classical expression −(1+logp) with *p* replaced by *f*. This explains why Theorem 1 returns a distribution of exponential form despite the entropy functional being built from two separate real fields.

Third, the same result follows from Wirtinger calculus. With $\partial /\partial f=\frac{1}{2}\left(\partial /\partial u-i\;\partial /\partial v\right)$ and $\Phi =\frac{1}{2}\left(f\log f+\bar{f\log f}\right)$, one finds $\partial \Phi /\partial f=\frac{1}{2}\left(1+\log f\right)$, and multiplying by two recovers (22). The agreement of the two routes is a check on the algebra rather than an independent derivation, since both rest on the same branch conventions.

### 3.4. The Complex Gibbs Distribution

**Theorem** **1** (Complex Gibbs distribution)**.** *A stationary point $f^{*}=u^{*}+iv^{*}$ of the Lagrangian (7) satisfies*

(24) $$\begin{array}{cc} \log|f^{*}\left(x\right)| & =-1-\lambda_{0}-\sum_{k=1}^{K}\alpha_{k}g_{k}\left(x\right), \end{array}$$

(25) $$\begin{array}{cc} \theta^{*}\left(x\right) & =\mu_{0}+\sum_{k=1}^{K}\beta_{k}g_{k}\left(x\right). \end{array}$$

*Defining complex Lagrange multipliers $\Lambda_{k}:=\alpha_{k}-i\beta_{k}\in C$ and the complex partition function*

$$Z\left(\Lambda \right)=\int_{-\infty }^{\infty }\exp\;\left(-\sum_{k=1}^{K}\Lambda_{k}g_{k}\left(x\right)\right)dx,$$

*the optimal CPDF is*

(26) $$f^{*}\left(x\right)=\frac{1}{Z(\Lambda )}\exp\;\left(-\sum_{k=1}^{K}\Lambda_{k}g_{k}\left(x\right)\right).$$

*The classical Gibbs distribution (3) is recovered when $\Lambda_{k}\in R$ for all k, which occurs precisely when all imaginary constraints vanish ($c_{k}^{\left(v\right)}=0$) and $\mu_{0}=0$.*

**Proof.**  Setting δL/δu=0 and δL/δv=0 yields (24) and (25) by substituting the functional derivatives (16) and (21). Combining,

$$f^{*}\left(x\right)=e^{-1-\lambda_{0}+i\mu_{0}}\exp\;\left(-\sum_{k}\left(\alpha_{k}-i\beta_{k}\right)g_{k}\left(x\right)\right)=e^{-1-\lambda_{0}+i\mu_{0}}\exp\;\left(-\sum_{k}\Lambda_{k}g_{k}\left(x\right)\right).$$

The normalization condition $\int f^{*}\;dx=1$ identifies the prefactor as 1/Z(Λ).    □

**Remark** **2** (Phase modulation)**.** *The complex Gibbs distribution (26) factorizes as*

$$f^{*}\left(x\right)=\frac{1}{Z(\Lambda )}\underset{\mathrm{classical}\;\mathrm{amplitude}}{\underset{︸}{e^{-\sum_{k}\alpha_{k}g_{k}\left(x\right)}}}\cdot \underset{\mathrm{phase}\;\mathrm{modulation}}{\underset{︸}{e^{i\sum_{k}\beta_{k}g_{k}\left(x\right)}}}.$$

*The imaginary multipliers $\beta_{k}$ introduce a spatially varying phase, modulating the classical Gibbs distribution without altering its functional form. For $\beta_{k}=0$ the phase modulation is trivial and the classical distribution is recovered.*

### 3.5. Boundedness, Existence and Optimality

Theorem 1 identifies a stationary point of Re(H[f]). Two further questions must be settled before the solution can be called a maximum The first concerns boundedness. The real part *u* is constrained by (D1) and by normalization, but the imaginary part *v* is bounded only through the constraints imposed on it. If those constraints leave any direction of *v* free, the entropy is unbounded above.

**Proposition** **2** (Unboundedness under incomplete constraints)**.** *Suppose the constraint functions ${\left\{g_{k}\right\}}_{k=1}^{K}$ do not span the space of functions on the support, so that there exists a real n with ∫ndx=0 and $\int g_{k}\;n\;dx=0$ for every k. Then Re(H[f]) is unbounded above on the set of complex densities satisfying (D1)–(D3) and the prescribed moments, and grows as Re(H)∼π2∥v∥1 along the corresponding direction.*

**Proof.**  Let $f_{a}=u+i\left(v+an\right)$. Every prescribed moment is unchanged by construction, and *u* is unchanged, so (CP1) continues to hold. As a→∞ one has $\left|f_{a}|∼a|n\right|$ and θ→±π/2 pointwise off the zero set of *n*. In

(27) Re(H[fa])=−∫ulog|fa|−(v+an)θdx,

the first term grows only logarithmically in *a* while the second grows linearly, giving Re(H[fa])∼π2a∥n∥1.    □

The resolution is contained in the formulation itself. The complex MaxEnt problem (6) prescribes complex moments $c_{k}=c_{k}^{\left(u\right)}+ic_{k}^{\left(v\right)}$, which fix the real *and* the imaginary projection of each constraint. When the family $\left\{g_{k}\right\}$ is complete in that sense the imaginary part is determined and the variational problem is well posed. Prescribing only the real moments and expecting the imaginary part to be selected variationally is ill posed. We state this as a requirement on the formulation.

The second question concerns curvature. Differentiating the entropy density Φ(u,v)=ulog|f|−vθ once more, using (16) and (21), gives

(28) $$\nabla^{2}\Phi =\frac{1}{{\left|f\right|}^{2}}\left(\begin{array}{cc} u & v\\ v & -u \end{array}\right),\;\mathrm{tr}\nabla^{2}\Phi =0,\;\det\nabla^{2}\Phi =-\frac{1}{{\left|f\right|}^{2}},$$

with eigenvalues ±1/|f|. The Hessian is therefore indefinite wherever f≠0, and so is that of Re(H), which is its negative. In the classical case the corresponding second derivative is $\partial^{2}\left(u\log u\right)/\partial u^{2}=1/u>0$, strictly convex, and it is that convexity which guarantees uniqueness of the classical Gibbs distribution.

(26) is a stationary point of Re(H[f]). Its status as a constrained maximum depends on the constraint set and on (CP1) restricting the admissible variations. We retain the name “maximum entropy” for the principle, as is standard, while using “stationarity” where the mathematical claim requires it. The situation is the same as in analytical mechanics, where the principle of least action is universally so called although the action is in general only stationary: beyond the kinetic focus it is a saddle point, and it is never a true maximum.

### 3.6. Dual Relations and Free Energy

**Proposition** **3** (Dual relations)**.** *The complex partition function Z(Λ) generates the prescribed moments via*

(29) $$c_{k}=-\frac{\partial \log Z}{\partial \Lambda_{k}},\;k=1,\ldots ,K.$$

*The complex entropy at the optimal distribution satisfies*

(30) $$H\left[f^{*}\right]=\log Z\left(\Lambda \right)+\sum_{k=1}^{K}\Lambda_{k}c_{k}\;\in \;C,$$

*so Re(H[f∗])=Re(logZ)+∑k(αkck(u)+βkck(v)).*

**Proof.**  Differentiate logZ(Λ) with respect to $\Lambda_{j}$ under the integral sign, justified by dominated convergence for *Z* finite and non-zero in a neighborhood of Λ, to obtain (29). Equation (30) follows from integrating $f^{*}\log f^{*}=f^{*}\left(-\log Z-\sum_{k}\Lambda_{k}g_{k}\right)$ and using $\int f^{*}\;dx=1$ and $\int g_{k}f^{*}\;dx=c_{k}$.    □

### 3.7. The (CP1) Admissibility Condition

A major difference from the classical approach is that not every choice of $\Lambda \in C^{K}$ produces a valid CPDF. Writing $\log Z\left(\Lambda \right)=\rho +i\phi_{Z}$, the real part of $f^{*}$ is

(31) Re(f∗(x))=e−ρ−∑kαkgk(x)cos−ϕZ+∑kβkgk(x).

Since the exponential factor is positive, (CP1) requires

(32) $$-\phi_{Z}+\sum_{k=1}^{K}\beta_{k}g_{k}\left(x\right)\;\in \;\left[-\frac{\pi }{2},\frac{\pi }{2}\right]\;\mathrm{for}\;\mathrm{all}\;x\in \mathrm{supp}\left(f^{*}\right).$$

This is the admissibility condition on the imaginary multipliers $\beta_{k}$. It defines a convex region in the space of imaginary multipliers whenever the constraint functions $g_{k}$ are bounded on the support.

The admissibility condition is the central structural difference between classical and complex MaxEnt, as Table 2 makes explicit. In the classical row of that table, the column “admissibility” reads “always satisfied”: since $\alpha_{k}\in R$ and Z(α)>0, the density $p^{*}\propto e^{-\sum \alpha_{k}g_{k}}$ is manifestly non-negative. In the complex row, the imaginary multipliers $\beta_{k}$ must be chosen so that the total phase $-\phi_{Z}+\sum_{k}\beta_{k}g_{k}\left(x\right)$ stays within (−π/2,π/2) everywhere on the support. This is a constraint on how large the imaginary Lagrange multipliers can be relative to the range of the constraint functions. For bounded $g_{k}$ on a compact support, the condition defines an open convex set in the $\left(\beta_{1},\ldots ,\beta_{K}\right)$-space, and the classical solution ($\beta_{k}=0$ for all *k*) always lies at its center, so the complex extension is a neighborhood of the classical solution in the space of valid multipliers.

When the admissibility condition fails, Re(f∗)<0 on some region of the support, meaning the proposed solution is not a CPDF. The interpretation is that the prescribed imaginary constraint $c^{\left(v\right)}$ is too large to be consistent with a non-negative-real-part density given the constraint functions $g_{k}$ and the real multipliers $\alpha_{k}$. This failure (or feature) has no analogue in classical MaxEnt, where the Gibbs distribution always exists for any finite $\alpha_{k}$.

The admissibility condition is therefore the boundary of the new inferential territory that complex MaxEnt opens: inside the boundary, the complex Gibbs distribution is a valid CPDF and the extra imaginary information can be incorporated; outside, the information is inconsistent with the (CP1) axiom and cannot be represented within the framework.

## 4. The Die Problem (Jaynes’ Brandeis Dice)

The classical die problem is the standard textbook application of Jaynes’ MaxEnt [1]. In the new formalism, a six-sided die assigns probabilities $p_{k}\in C$ to face k∈{1,…,6}. The complex probability measure on Ω={1,…,6} satisfies $p_{k}=u_{k}+iv_{k}$ with $u_{k}\geq 0$, $\sum u_{k}=1$, and $\sum v_{k}=0$. The single constraint function is g(k)=k (face value), and the prescribed complex moment is $c=c^{\left(u\right)}+ic^{\left(v\right)}\in C$:

(33) $$\sum_{k=1}^{6}k\;u_{k}=c^{\left(u\right)}\;\mathrm{and}\;\sum_{k=1}^{6}k\;v_{k}=c^{\left(v\right)}.$$

### 4.1. Case 1: The Classical Die (Kolmogorov–Jaynes Baseline)

A classical six-sided die produces face values k=1,…,6. The only information available is the mean face value, $c^{\left(u\right)}=\mu \in R$. Applying MaxEnt with the single real constraint $\sum_{k}k\;p_{k}=\mu$ and the normalization $\sum_{k}p_{k}=1$ gives the exponential family

(34) $$p_{k}^{\mathrm{class}}=\frac{e^{-\alpha k}}{Z_{\mathrm{class}}\left(\alpha \right)},\;Z_{\mathrm{class}}\left(\alpha \right)=\sum_{k=1}^{6}e^{-\alpha k},$$

where α∈R is the Lagrange multiplier determined by $-\partial \log Z_{\mathrm{class}}/\partial \alpha =\mu$.

For a fair die, the natural mean is μ=7/2=3.5, which carries no information beyond the range of possible values. MaxEnt returns the uniform distribution: α=0 gives $p_{k}^{\mathrm{class}}=1/6$ for all *k* and $Z_{\mathrm{class}}\left(0\right)=6$. This is the unique distribution that is maximally non-committal about the face value given only that the mean is 3.5.

For a loaded die (μ≠3.5), the sign of the Gibbs parameter α is determined entirely by the direction of loading: α<0 when μ>3.5 and α>0 when μ<3.5. The case μ≈4.33 gives α=−0.30, producing the monotonically increasing distribution $p_{k}^{\mathrm{class}}\propto e^{0.30k}$, in which face 6 carries approximately $e^{1.5}\approx 4.48$ times the probability of face 1. The mode is at the boundary face 6 in consequence. What is less immediately obvious is that this boundary-mode property holds for *any* mean strictly greater than 3.5, no matter how small the excess. A die with μ=3.51, whose mean is barely distinguishable from the symmetric value, nonetheless produces a distribution that places its entire maximum at k=6, well away from the mean. This is not a numerical accident but a structural consequence of the exponential family on a bounded integer support: any non-zero α renders the distribution strictly monotone, so the mode cannot be interior. The result is formally correct, but it exposes a genuine limitation of classical MaxEnt as a tool for encoding distributional shape, since no mechanism exists within the framework to place the mode near the mean without prescribing additional moment constraints.

In both cases, fair and loaded, all probabilities are real, non-negative, and sum to one. The imaginary part of every $p_{k}$ is zero by construction; there is no additional degree of freedom. The following two cases show what changes when complex constraints are admitted.

### 4.2. Case 2: Complex MaxEnt, Fair Real Mean

Retain $c^{\left(u\right)}=7/2$, the same real constraint as the classical fair die, but add an imaginary constraint $c^{\left(v\right)}\neq 0$. Set α=0 and β=π/12, so Λ=−iπ/12. The complex Gibbs distribution is   

(35) $$p_{k}^{*}=\frac{e^{i\pi k/12}}{Z(-i\pi /12)},$$

and the partition function is the geometric sum

(36) $$Z\left(-i\pi /12\right)=\sum_{k=1}^{6}e^{i\pi k/12}\approx 3.2978+4.2978i.$$

Since $|e^{i\pi k/12}|=1$ for all *k*, every face carries the same modulus $|p_{k}^{*}|=1/|Z|\approx 0.1846$, and the arguments advance by $15^{\circ }$ per face:

(37) $$\arg\left(p_{k}^{*}\right)=15^{\circ }\left(k-3.5\right),\;k=1,\ldots ,6.$$

The admissibility condition (32) is satisfied: $|\arg\left(p_{k}^{*}\right)|\leq 37.5^{\circ }<90^{\circ }$ for all *k*. The explicit values are given in Table 3.

Two consequences of the imaginary constraint are worth noting explicitly.

First, the real distribution $\left\{u_{k}^{*}\right\}$ is not uniform, even though the real constraint is the same as for a fair die. The imaginary constraint $c^{\left(v\right)}$ has redistributed the observable probability mass toward the central faces (3 and 4), producing $u_{3}^{*}=u_{4}^{*}\approx 0.183>1/6$, while the extreme faces are suppressed: $u_{1}^{*}=u_{6}^{*}\approx 0.147<1/6$. This is the fundamental departure from classical MaxEnt: additional information, encoded in the imaginary part, changes the observable distribution.

Second, the complex mean is

(38) $$c=\sum_{k=1}^{6}k\;p_{k}^{*}\approx 3.500+0.798i,$$

so $c^{\left(u\right)}=3.5$, the real mean is preserved, and $c^{\left(v\right)}\approx 0.798$, the imaginary moment being the prescribed constraint fixed by the choice β=π/12.

### 4.3. Case 3: Loaded Die

Load the die by choosing α=−0.30 in the real part, so that the classical Gibbs distribution assigns monotonically more probability to higher faces. The classical mean at α=−0.30 and β=0 is $\mu_{\mathrm{class}}=\sum_{k}k\;e^{0.30k}/Z_{\mathrm{class}}\approx 4.33$, reflecting a bias toward faces 5 and 6. Adding the same imaginary multiplier β=π/12 as in Case 2 gives Λ=−0.30−iπ/12 and the complex Gibbs distribution

(39) $$p_{k}^{*}=\frac{e^{-\Lambda k}}{Z(\Lambda )}=\frac{e^{0.30k}\;e^{i\pi k/12}}{Z(\Lambda )},\;Z\left(\Lambda \right)=\sum_{k=1}^{6}e^{-\Lambda k}\approx 7.441+16.254i.$$

The factor $e^{0.30k}$ is the classical loading, increasing with face value; the factor $e^{i\pi k/12}$ is the same phase modulation as in Case 2. Because α≠0, the equal-modulus property of Case 2 is broken: $|p_{k}^{*}|$ now varies with *k*, ranging from 0.076 at face 1 to 0.338 at face 6. Table 4 gives all values.

A quick verification shows that $\sum u_{k}^{*}=1.000$ and $\sum v_{k}^{*}=0$ to machine precision. All Re(pk∗)>0, so (CP1) holds. The complex mean is $c=\sum_{k}k\;p_{k}^{*}\approx 4.423+0.664i$, so $c^{\left(u\right)}\approx 4.42$, the real mean biased high as intended, and $c^{\left(v\right)}\approx 0.664$, the imaginary constraint produced by β=π/12.

The admissibility condition also holds. The argument of Z(Λ) is $\phi_{Z}\approx 65.4^{\circ }$. The total phase $-\phi_{Z}+\beta k$ ranges from $-50.4^{\circ }$ at face 1 to $+24.6^{\circ }$ at face 6, all strictly within $\left(-90^{\circ },90^{\circ }\right)$. The distribution is admissible.

### 4.4. Mode Structure and the Imaginary Constraint

A principal advantage of the complex MaxEnt framework is that it provides, through the imaginary Lagrange multiplier β, control over the mode of the resulting distribution without adding any classical moment constraint. Classical MaxEnt seeks the most non-committal probability distribution consistent with the prescribed constraints; the present extension preserves that goal while enlarging the family of achievable distributions to include those whose mode lies in the interior of the support, a property that the classical exponential family cannot exhibit for any mean other than the symmetric point.

#### 4.4.1. The Classical Mode Problem

For the classical Gibbs distribution $p_{k}^{\mathrm{class}}\propto e^{-\alpha k}$ on {1,…,6}, the function $k\mapsto e^{-\alpha k}$ is strictly monotone for any α≠0: decreasing for α>0 and increasing for α<0. The mode is therefore always at a boundary face, k=1 when α>0 (mean below 3.5) and k=6 when α<0 (mean above 3.5). The only non-boundary mode is the uniform distribution at α=0, which has no unique mode. For any prescribed mean μ≠3.5, the classical MaxEnt solution concentrates its maximum at an extreme face regardless of how close μ is to 3.5.

This is not a deficiency of the MaxEnt principle itself, for the exponential family is the correct solution to the variational problem as stated. It is rather a structural consequence of the linear constraint function g(k)=k combined with the exponential form. Unfortunately, there is no additional freedom in the classical framework to shape the mode position independently of the mean.

#### 4.4.2. Mode Formula for Complex MaxEnt

In contrast, write the complex Gibbs distribution as $p_{k}^{*}=\left|p_{k}^{*}\right|e^{i\theta_{k}}$ where $|p_{k}^{*}|=e^{-\alpha k}/\left|Z\left(\Lambda \right)\right|$ and $\theta_{k}=-\phi_{Z}+\beta k$, with $\phi_{Z}=\arg\left(Z\left(\Lambda \right)\right)$. The observable probability is $u_{k}^{*}=\left|p_{k}^{*}\right|\cos\theta_{k}$. Treating *k* as continuous and differentiating,

$$\frac{du_{k}^{*}}{dk}=\frac{e^{-\alpha k}}{\left|Z\right|}\left(-\alpha \cos\theta_{k}-\beta \sin\theta_{k}\right)=0$$

at the mode $k^{*}$, which gives $\tan\theta_{k^{*}}=-\alpha /\beta$ and therefore

(40) $$k^{*}=\frac{\phi_{Z}\left(\Lambda \right)+\arctan\left(-\alpha /\beta \right)}{\beta }.$$

The mode depends on both the real multiplier α, through $\phi_{Z}$ and the arctangent term, and the imaginary multiplier β, directly in the denominator and through $\phi_{Z}$. When α=0, Equation (40) simplifies to $k^{*}=\phi_{Z}\left(-i\beta \right)/\beta$.

#### 4.4.3. Exact Result for the Fair Die

The simplest case is the fair die.

**Proposition** **4** (Mode equals mean for the unloaded die)**.** *For α=0 and any β∈(0,π/5), the complex Gibbs distribution with multiplier Λ=−iβ satisfies the admissibility condition and has mode $k^{*}=3.5=c^{\left(u\right)}$ exactly.*

**Proof.**  Applying the geometric series to $Z\left(-i\beta \right)=\sum_{k=1}^{6}e^{i\beta k}$,

(41) $$Z\left(-i\beta \right)=e^{i\beta }\cdot \frac{1-e^{6i\beta }}{1-e^{i\beta }}=e^{3.5i\beta }\cdot \frac{\sin3\beta }{\sin(\beta /2)}.$$

Since sin3β/sin(β/2)>0 for β∈(0,π/3), the factor $e^{3.5i\beta }$ carries the entire argument: $\phi_{Z}=3.5\beta$. Substituting into (40) with α=0 gives $k^{*}=3.5\beta /\beta =3.5$. For admissibility, the phase at face *k* is $-\phi_{Z}+\beta k=\beta \left(k-3.5\right)$, ranging from −2.5β at k=1 to +2.5β at k=6. The condition |2.5β|<π/2 is equivalent to β<π/5, which is satisfied throughout the stated interval. Since π/5<π/3, Equation (41) also holds.    □

Proposition 4 establishes that the imaginary constraint corrects the boundary-mode limitation of classical MaxEnt for the unloaded die: for any admissible β, the mode of the observable distribution is exactly at the midpoint of the support, coinciding with the real mean $c^{\left(u\right)}=3.5$. The result is independent of the size of β within the admissible range, holding equally at $\beta =5^{\circ }$ and $\beta =35^{\circ }$. The die example with $\beta =\pi /12=15^{\circ }$ (Case 2) is one instance of this family.

#### 4.4.4. The Loaded Die and the Role of (CP1)

For α≠0, Equation (40) no longer simplifies, and the mode location depends on both α and β. For the loaded die with α=−0.30 and β=π/12, Equation (40) gives $k^{*}\approx 7.6$, outside the support {1,…,6}, so the mode remains at k=6. The imaginary constraint β=π/12 is insufficient to overcome the loading. A natural question is whether a larger β could shift the mode to an interior location.

Numerical computation shows that the mode of $\left\{u_{k}^{*}\right\}$ leaves k=6 at $\beta \approx 28.8^{\circ }$. At that value; however, the phase at k=1 is $-99.4^{\circ }$, violating the admissibility condition $|\theta_{k}|<\pi /2$. The loaded die with α=−0.30 therefore admits no admissible imaginary constraint that produces an interior mode. (CP1) is precisely the constraint that prevents mode correction when the loading is too strong.

This is a strong structural result. The admissibility condition (32), which arose from requiring Re(f∗)≥0 everywhere, acts as a barrier that limits how much the imaginary multiplier can reshape the observable distribution. For the fair die, this barrier is wide enough to permit full mode correction across the entire admissible range. For the loaded die with |α| large enough, the barrier is too tight: the phase rotation needed to create an interior mode pushes some faces below the real axis, violating (CP1).

It is worth separating what in this failure is intrinsic to the axiom from what merely reflects the normalization. The phase at state *k* is $\theta_{k}=-\phi_{Z}+\beta k$, so the total excursion across the support is β(N−1), which does not involve $\phi_{Z}$ and is therefore independent of the normalization. The partition function enters only through $\phi_{Z}=\arg Z\left(\Lambda \right)$, which translates the phase window without widening it. Condition (CP1) requires that window to fit inside [−π/2,π/2], hence β≤π/(N−1), which for N=6 is $36^{\circ }$. For α=−0.30 the mode leaves face 6 only at $\beta \approx 28.8^{\circ }$, at which point the phase at face 1 has reached $-99.4^{\circ }$. No choice of normalization recovers the missing $9.4^{\circ }$, because the width of the excursion does not depend on it. The obstruction is thus a property of (CP1) together with the geometry of the support and the range of the constraint function, and not of the $L^{1}$ normalization structure.

The general form of the statement delimits where the framework has room to act. On an unbounded support with polynomial constraint functions the imaginary part of the Gibbs exponent is an unbounded polynomial, so the phase necessarily leaves [−π/2,π/2] and (CP1) forces every imaginary multiplier to vanish. Genuine freedom exists only on bounded or discrete supports. The loaded die is an instance of that bound biting rather than of a defect peculiar to this example, and the same fact governs the numerical study of Appendix A.

The two cases together reveal the role of β as a *constrained shape parameter*: it can redistribute observable probability mass from the boundaries toward the interior, but only within the region permitted by the non-negativity axiom. Classical MaxEnt has no such parameter. The complex framework not only provides it but also determines its range through (CP1).

### 4.5. Mode Specification: The Impossible Classical Problem

The results above suggest a problem that classical MaxEnt cannot solve but complex MaxEnt can: prescribing simultaneously the mean μ and the mode $k^{*}$ of the distribution. The mean is a linear functional of *p* and can be encoded as a classical constraint. The mode is the argmax of *p*, a nonlinear functional that cannot be expressed as any expectation $\sum_{k}g\left(k\right)\;p_{k}=c$ for any *g*.

Setting the mean and mode simultaneously in the complex MaxEnt framework reduces to solving the two-equation system

(42) $$\mu =-\frac{\partial \log Z(\Lambda )}{\partial \alpha },\;k^{*}=\frac{\phi_{Z}\left(\Lambda \right)+\arctan\left(-\alpha /\beta \right)}{\beta },$$

in two unknowns (α,β). The admissibility condition (32) constrains the solution to lie within the right half-plane Re(Λ)>0.

The die example illustrates how this formalism can be applied. Table 5 summarizes, from exhaustive numerical search over the admissible parameter space of the case, which $\left(\mu ,k^{*}\right)$ pairs are accessible to classical MaxEnt (only boundary modes), to complex MaxEnt only (interior modes, marked †), or to both frameworks.

An illustrative example is μ=4.0 with mode at k=5. Classical MaxEnt at μ=4.0 gives $\alpha_{\mathrm{cl}}=-0.1746$, β=0, and $u_{k}^{\mathrm{cl}}=\left(0.103,\;0.123,\;0.146,\;0.174,\;0.207,\;0.247\right)$, with mode at k=6. Solving system (42) for μ=4.0 and $k^{*}=5$ yields Λ=−0.1378−0.3501i and

$$u_{k}^{*}=\left(0.071,\;0.123,\;0.172,\;0.208,\;0.222,\;0.205\right),$$

with mode at k=5 and real mean $c^{\left(u\right)}=4.000$. The admissibility condition is satisfied, all phases lying within $\left(-90^{\circ },90^{\circ }\right)$. The imaginary constraint is $c^{\left(v\right)}=1.057$, encoding directional information about the sub-population structure.

Note that the classical Shannon entropy of the complex solution (1.734) is slightly below that of the classical Gibbs (1.749), reflecting the additional information encoded in the imaginary constraint. The complex entropy Re(H[p∗])=1.942 exceeds the classical maximum, because it optimizes over a strictly larger family of distributions.

### 4.6. Geometric Representation in the Complex Plane

Figure 1 displays the six complex probabilities $p_{k}^{*}\in C$ for both Case 2 (fair die) and Case 3 (loaded die) as vectors in the complex plane, with the argument $\theta_{k}^{*}=\arg\left(p_{k}^{*}\right)$ of each vector marked explicitly.

In Case 2, panel b, all six vectors have the same modulus $|p_{k}^{*}|=1/|Z|\approx 0.1846$, so their tips lie on the dashed circle. The arguments advance by exactly $15^{\circ }$ per face, from $\theta_{1}^{*}=-37.5^{\circ }$ to $\theta_{6}^{*}=+37.5^{\circ }$. The observable classical probability $u_{k}^{*}=\left|p_{k}^{*}\right|\cos\theta_{k}^{*}$ is thus largest for the central faces, where |θ| is smallest, and smallest for the extreme faces. The imaginary part $v_{k}^{*}=\left|p_{k}^{*}\right|\sin\theta_{k}^{*}$ is antisymmetric, encoding the imaginary constraint $c^{\left(v\right)}\approx 0.798$.

In Case 3, panel c, the loading α=−0.30 breaks the equal-modulus property: the vectors grow in length from $|p_{1}^{*}|\approx 0.076$ to $|p_{6}^{*}|\approx 0.338$, reflecting the real bias toward higher faces. The imaginary constraint β=π/12 still rotates each vector, but by an angle that now interacts with the variable moduli: face 1 has the largest negative angle, $-50.4^{\circ }$, because its small modulus amplifies the relative effect of the rotation. All vectors lie in the open right half-plane, consistent with (CP1).

Two structural observations emerge from the three cases. In Case 2, the same real constraint $c^{\left(u\right)}=3.5$ that classically forces the uniform distribution produces a bell-shaped real distribution when combined with $c^{\left(v\right)}\approx 0.798$. The imaginary information acts on the observable probabilities, concentrating mass toward the central faces. The mechanism is algebraic: the phase modulation $e^{i\beta k}$ in the numerator of (35), after normalization by the complex partition function Z(Λ)∈C, rotates the real parts of the $p_{k}^{*}$ away from the uniform distribution. In other words, the imaginary constraint changes the observable distribution.

In both Cases 2 and 3, the imaginary parts $v_{k}^{*}$ are antisymmetric: negative for small face values, positive for large ones. The imaginary constraint $c^{\left(v\right)}>0$ prescribes that there is more imaginary probability mass at high faces than at low faces, a form of directional information with no counterpart in classical probability. This information is not accessible to a classical observer, who measures only Re(pk∗), but it shapes the observable distribution in a measurable way. That is, the imaginary part encodes a directional signal.

## 5. Conclusions

The paper has proposed and developed a maximum entropy principle for complex probability measures. The key steps were the following.

Youssef’s framework, while sufficient to derive quantum mechanics from probabilistic axioms, does not admit a natural MaxEnt extension. Rodriguez’s Clifford extension [19] broadens Youssef’s framework to all Clifford algebras, recovering quaternionic and spinorial quantum mechanics as special cases, but inherits the same structural obstacle: the $L^{2}$ (Born-rule) normalization is incompatible with the $L^{1}$ normalization assumed by Shannon entropy, so no canonical MaxEnt principle exists within either framework.

The new framework, which replaces non-negativity with the real-part condition Re(P(A))≥0 and retains the linear normalization ∫f(x)dx=1 of an $L^{1}$ density, admits a natural MaxEnt principle. The real part of the complex Shannon entropy is a well-posed real functional. Its Euler–Lagrange equations, derived by treating the real and imaginary parts of the density as independent variational variables, produce the complex Gibbs distribution $f^{*}=e^{-\sum \Lambda_{k}g_{k}}/Z\left(\Lambda \right)$ with complex Lagrange multipliers $\Lambda_{k}\in C$. The classical Gibbs distribution is recovered when the imaginary parts of the multipliers vanish.

The die examples demonstrate the central qualitative consequence: adding an imaginary constraint to a classical constraint changes the observable real-part distribution, concentrating mass toward the central faces in the fair-die case and modifying the loading profile in the loaded-die case (Figure 2). The imaginary constraint carries directional information, invisible to classical measurement, that reshapes the observable distribution in a systematic way determined by the admissibility condition and the structure of the constraint functions.

Two questions remain open. The first, and the more fundamental one, is the operational meaning of the imaginary constraint $c^{\left(v\right)}$. No sequence of classical observations on the die can distinguish two distributions with the same real mean $c^{\left(u\right)}$ but different imaginary moments $c^{\left(v\right)}$, yet the imaginary constraint shapes the observable distribution through the complex Gibbs formula. Three candidate interpretations point in different directions.

The first is a Hahn–Jordan decomposition: the imaginary part $v_{k}^{*}=q_{k}^{\left(+\right)}-q_{k}^{\left(-\right)}$ decomposes into two non-negative parts corresponding to two latent sub-populations, and $c^{\left(v\right)}$ is proportional to the difference in expected face value between them. For the canonical case Λ=−0.1378−0.3501i (μ=4.0, mode $k^{*}=5$), the high sub-population concentrates on faces 5 and 6 (${\bar{k}}^{+}=5.64$) and the low sub-population on faces 1 to 3 (${\bar{k}}^{-}=1.78$), with $c^{\left(v\right)}=m\left({\bar{k}}^{+}-{\bar{k}}^{-}\right)=0.274\times 3.86=1.057$.

The second is an orthogonal measurement interpretation: classical measurements access only the simplex of real distributions; the imaginary part lies in the orthogonal complement of the constant function in $R^{N}$, inaccessible to any frequency-based observation. The imaginary constraint is a coordinate in this orthogonal complement, representing prior knowledge about the system’s internal structure that has no observable footprint. As β increases from 0 to π/5 at fixed μ=4.0, the pair $\left(H^{\mathrm{cl}},c^{\left(v\right)}\right)$ traces a curve from the classical point (1.749,0) to (1.581,1.929), with the mode shifting from k=6 to k=4.

The third connects to temporal antisymmetry: for a stationary Markov chain with marginal $u_{k}$, the probability current $J_{k,k+1}$ between adjacent states vanishes at equilibrium (detailed balance) and is non-zero out of equilibrium. The bond-averaged imaginary density $(v_{k}^{*}+v_{k+1}^{*})/2$ is proportional to $J_{k,k+1}$: a positive imaginary constraint $c^{\left(v\right)}>0$ corresponds to a net probability current flowing from low to high faces, encoding the observer’s prior knowledge that the system is not at equilibrium and has a preferred direction of dynamics.

These three interpretations will be developed in a subsequent paper, together with three speculative directions: linear response, entropic Bayesian updating, and the connection to hidden variables.

The second open question is one of convexity. One can ask is the complex Gibbs distribution is the unique maximum of Re(H[f]), or if could there be other stationary points. This is a question about the functional (5) under the (CP1) constraint (D1). Section 3.5 shows the answer is negative: the Hessian of the entropy density is traceless with negative determinant, hence indefinite, so the complex Gibbs distribution is a stationary point rather than a demonstrated maximum, and Re(H) is unbounded above whenever the constraint set leaves a direction of the imaginary part free. What remains open is narrower, namely whether that stationary point is the unique constrained maximum once the imaginary moments are prescribed and (CP1) is imposed.

## Figures and Tables

**Figure 1:**
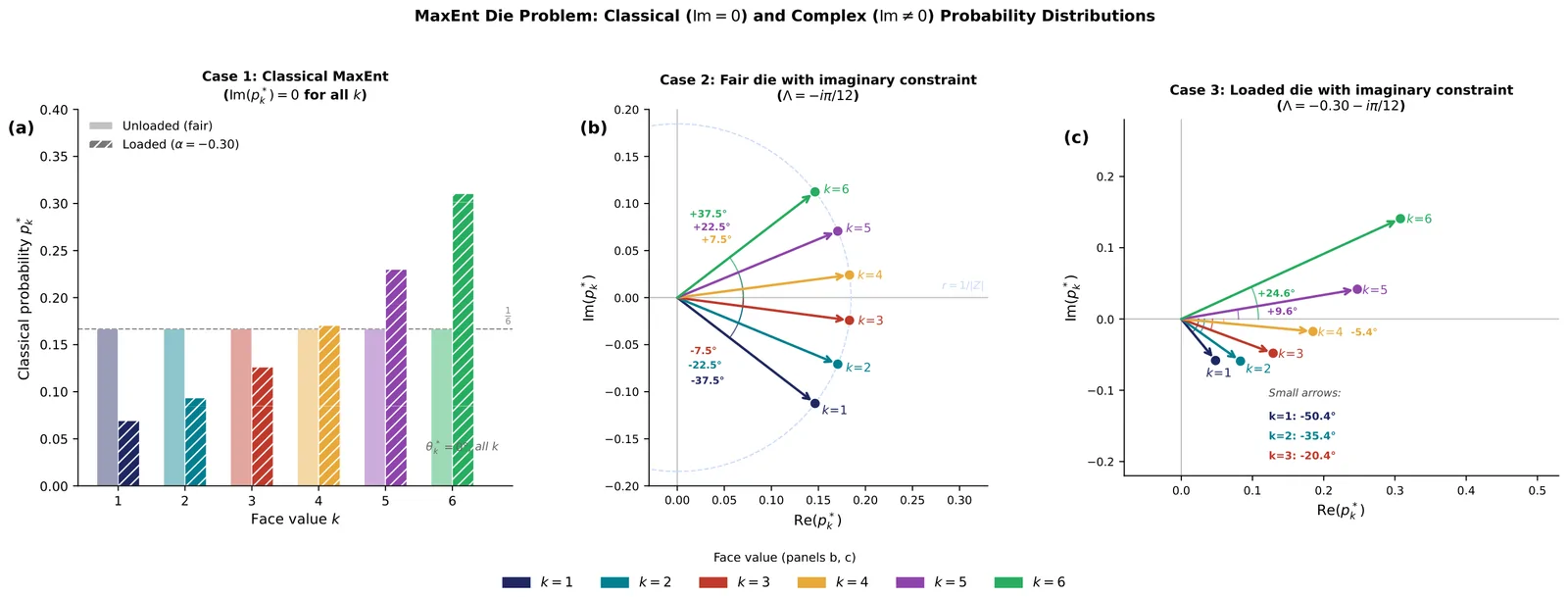
Three cases of the MaxEnt die problem. (**a**) Case 1, classical MaxEnt with real-valued probabilities (β=0, $\theta_{k}^{*}=0^{\circ }$ for all *k*). The *x*-axis gives the face value, the *y*-axis the classical probability $p_{k}^{*}$. Solid, lighter bars show the unloaded (fair) die, where MaxEnt returns the uniform distribution $p_{k}^{*}=1/6$ for all *k*, matching the dashed reference line. Hatched bars show the loaded die (α=−0.30), where the exponential family assigns monotonically increasing probabilities from k=1 to k=6. In both classical cases, all probability mass lies on the positive real axis of the complex plane. (**b**) Case 2, fair die with imaginary constraint (Λ=−iπ/12). All six complex probabilities lie on the dashed circle of radius 1/|Z|≈0.1846; their arguments advance linearly at $15^{\circ }$ per face. The horizontal projection of each vector, uk∗=Re(pk∗), is the observable probability and differs from the uniform distribution despite the same real constraint. (**c**) Case 3, loaded die with imaginary constraint (Λ=−0.30−iπ/12). The real loading breaks the equal-modulus property while the imaginary multiplier rotates each vector off the real axis; the three smallest arrows are annotated in the inset. In panels (**b**,**c**), all vectors lie in the open right half-plane, confirming (CP1).

**Figure 2:**
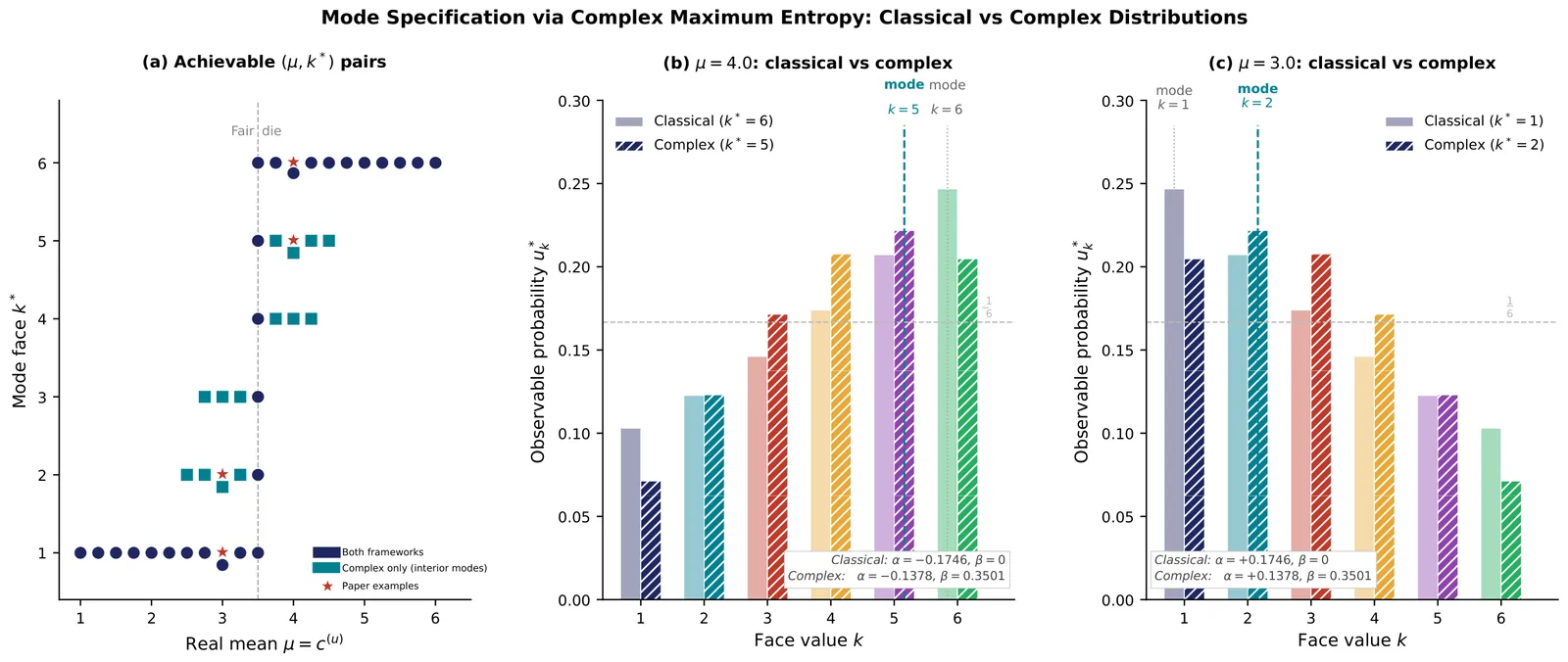
Mode specification via complex maximum entropy. (**a**) Achievable $\left(\mu ,k^{*}\right)$ pairs on a six-sided die. Navy circles: accessible to both frameworks. Teal squares: accessible to the complex framework only, that is the interior modes. Stars mark the two worked examples. No third category appears because none can exist: the classical solution is the case β=0, which is always admissible, so every classically achievable pair is also achievable in the complex framework and the classical set is a subset of the complex one. (**b**) Distributions at μ=4.0; classical Gibbs, monotone with mode k=6, versus complex MaxEnt (Λ=−0.1378−0.3501i, mode k=5), both at the same real mean. (**c**) Symmetric counterpart at μ=3.0; classical mode k=1 versus complex mode k=2. The symmetric Lagrange multiplier pair (α,β)=(±0.1378,0.3501) produces exact reflections.

**Table 1:** Comparison of the three probability frameworks. The new framework is intermediate: strictly more general than Kolmogorov (complex values) and strictly more constrained than Youssef or Rodriguez ($L^{1}$ normalization, real-part positivity). MaxEnt is naturally defined only in the first two columns; the third column has no canonical entropy functional compatible with its $L^{2}$ normalization.

Property	Kolmogorov	This Work	Youssef (1994)/Rodriguez (1998)
Axiom system	Measure theory	Measure theory, extended	Cox’s Bayesian axioms
Range of *P*	[0,1]⊂R	C, Re(P)≥0	C (Youssef) or Clifford algebra *G* (Rodriguez); unconstrained
Non-negativity	P(A)≥0	Re(P(A))≥0	None
Normalization	P(Ω)=1 ($L^{1}$)	P(Ω)=1 ($L^{1}$)	$\sum |P_{k}{|}^{2}=1$ ($L^{2}$, Born norm)
Observable prob.	P(A)	Re(P(A))	${\left|P\left(A\right)\right|}^{2}$ (Born rule)
Shannon entropy	H=−∫plogpdx	Re(H) well-posed	Not naturally defined
MaxEnt solution	Classical Gibbs	Complex Gibbs	No canonical solution
Recovers QM	No	Boundary limit (Λ→iR)	Yes, interior (Youssef); all signatures (Rodriguez)

**Table 2:** Classical Jaynes MaxEnt versus its complex extension. The complex formulation strictly generalizes the classical one: setting Im(Λk)=0 and $c_{k}^{\left(v\right)}=0$ for all *k* recovers the classical Gibbs distribution and all classical relations exactly.

Quantity	Classical MaxEnt	Complex MaxEnt
Density	p≥0, ∫pdx=1	f=u+iv, Re(f)≥0, ∫fdx=1
Entropy	−∫plogpdx∈R	Re(−∫flogfdx)∈R
Constraints	$\int g_{k}p\;dx=c_{k}\in R$	$\int g_{k}f\;dx=c_{k}\in C$
Multipliers	$\alpha_{k}\in R$	$\Lambda_{k}=\alpha_{k}-i\beta_{k}\in C$
Gibbs density	$e^{-\sum \alpha_{k}g_{k}}/Z$	$e^{-\sum \Lambda_{k}g_{k}}/Z\left(\Lambda \right)$
Partition function	$Z\in R_{>0}$	Z(Λ)∈C∖{0}
Dual relation	$-\partial \log Z/\partial \alpha_{k}=c_{k}$	$-\partial \log Z/\partial \Lambda_{k}=c_{k}$
Admissibility	Always satisfied	Condition (32)
Recovery	$\Lambda_{k}\in R$ and $c_{k}^{\left(v\right)}=0$ give the classical case	

**Table 3:** Classical MaxEnt ($u_{k}^{\mathrm{class}}=1/6$) versus complex MaxEnt ($p_{k}^{*}=u_{k}^{*}+iv_{k}^{*}$) for Λ=−iπ/12, with the same real constraint $c^{\left(u\right)}=3.5$. All faces share the same modulus; the distribution of probability in C is encoded in the linearly spaced phases $\theta_{k}^{*}$.

*k*	$u_{k}^{\mathrm{class}}$	$u_{k}^{*}$	$v_{k}^{*}$	$\theta_{k}^{*}$	$|p_{k}^{*}|$
1	$0.1\bar{6}$	0.1465	−0.1124	$-37.5^{\circ }$	0.1846
2	$0.1\bar{6}$	0.1706	−0.0707	$-22.5^{\circ }$	0.1846
3	$0.1\bar{6}$	0.1830	−0.0241	$-7.5^{\circ }$	0.1846
4	$0.1\bar{6}$	0.1830	+0.0241	$+7.5^{\circ }$	0.1846
5	$0.1\bar{6}$	0.1706	+0.0707	$+22.5^{\circ }$	0.1846
6	$0.1\bar{6}$	0.1465	+0.1124	$+37.5^{\circ }$	0.1846

**Table 4:** Complex MaxEnt distribution for the loaded die (Λ=−0.30−iπ/12). The column $u_{k}^{\mathrm{class}}$ gives the classical Gibbs distribution for α=−0.30, β=0, i.e., the same real loading with no imaginary constraint. The imaginary constraint shifts observable probability from the extreme faces to the intermediate ones and introduces antisymmetric imaginary parts.

*k*	$u_{k}^{*}$	$v_{k}^{*}$	$\theta_{k}^{*}$	$|p_{k}^{*}|$	$u_{k}^{\mathrm{class}}$
1	0.0481	−0.0582	$-50.4^{\circ }$	0.0755	0.0693
2	0.0831	−0.0590	$-35.4^{\circ }$	0.1019	0.0935
3	0.1290	−0.0480	$-20.4^{\circ }$	0.1376	0.1262
4	0.1849	−0.0175	$-5.4^{\circ }$	0.1857	0.1704
5	0.2472	+0.0418	$+9.6^{\circ }$	0.2507	0.2300
6	0.3077	+0.1409	$+24.6^{\circ }$	0.3384	0.3105

**Table 5:** Achievable mode faces $k^{*}$ at each mean μ for classical and complex MaxEnt on {1,…,6} with constraint g(k)=k. Pairs marked † are accessible only to the complex framework within the admissible region. Classical MaxEnt achieves only the boundary modes k=1 (for μ<3.5) and k=6 (for μ>3.5), or the entire set trivially for μ=3.5 (uniform distribution, no unique mode).

μ	Classical $k^{*}$	Complex $k^{*}$ (Admissible)
1.0–2.25	1	1
2.5–3.25	1	1, 2 ^†^
3.0	1	1, 2 ^†^, 3 ^†^
3.5	all (uniform)	1 ^†^, 2 ^†^, 3 ^†^, 4 ^†^, 5 ^†^, 6 ^†^
3.75–4.0	6	4 ^†^, 5 ^†^, 6
4.25–4.5	6	5 ^†^, 6
4.75–6.0	6	6

**Figure A1:**
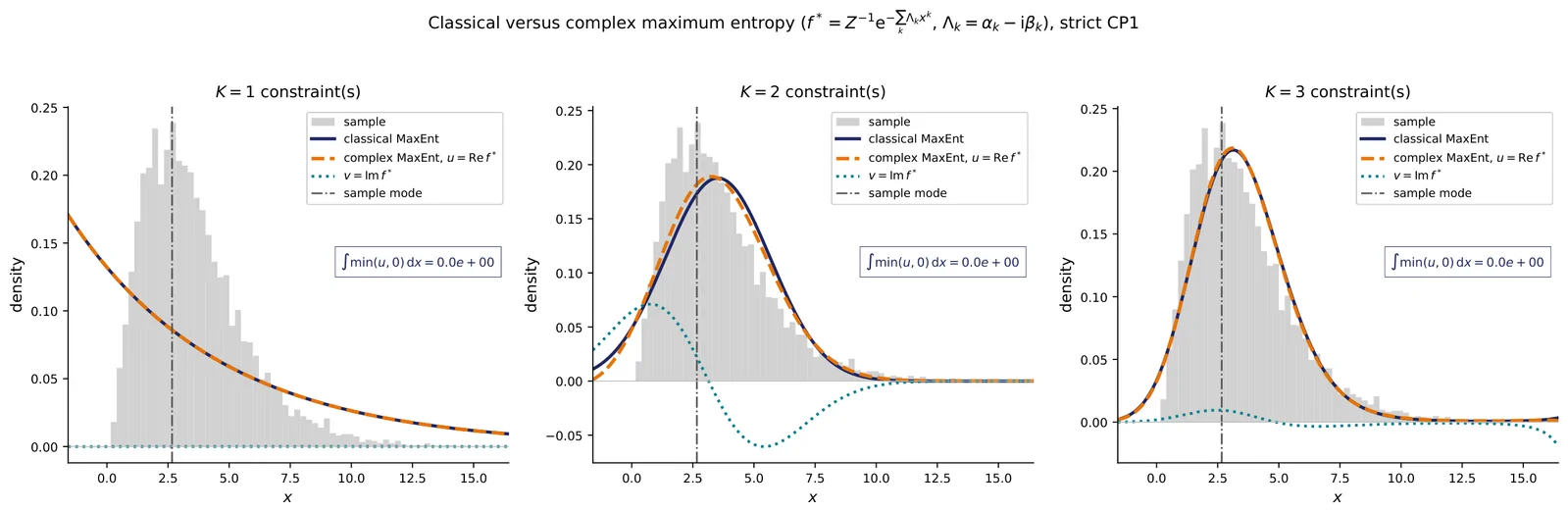
Classical and complex maximum entropy distributions fitted to a synthetic sample of n=5000 observations from a Gamma distribution with shape a=3 and scale s=1.2 (mean μ=3.6, mode $x^{†}=2.4$, variance $\sigma^{2}=4.32$), shown for K=1 (**left**), K=2 (**center**), and K=3 (**right**) power-moment constraints $g_{k}\left(x\right)=x^{k}$, k=1,…,K. The grey histogram shows the empirical density. The solid navy curve is the classical MaxEnt distribution $p^{*}\left(x\right)\propto \exp\left(-\sum_{k}\lambda_{k}x^{k}\right)$, with real multipliers λ determined by the sample moments. The dashed orange curve is the observable complex MaxEnt distribution u∗(x)=Ref∗(x), with complex multipliers $\Lambda_{k}=\alpha_{k}-i\beta_{k}$ obtained by the mode-relaxation routine fit_mode: the imaginary multipliers β are searched so that $\arg\max_{x}u^{*}\left(x\right)$ coincides with the KDE-estimated sample mode, the dash-dot vertical line at $x^{†}\approx 2.4$. The dotted teal curve is the imaginary part v∗(x)=Imf∗(x), shown on the same density axis; it integrates to zero by the normalization axiom (CP2) and encodes the sub-population structure of the imaginary constraint. The figure has been generated with the admissibility tolerance set to zero, so that (CP1) is enforced strictly, and the integrated negative part $\int \min(u^{*},0)\;dx$ is reported in each panel; it vanishes to machine precision in all three. Under strict enforcement the outcome is restrictive. For K=1 the search returns $\max_{k}\left|\beta_{k}\right|=6.9\times 10^{-5}$, so the complex and classical solutions coincide and no mode correction occurs: with a single linear constraint the phase $\beta_{1}x$ is unbounded on the sample support, and (CP1) then forces $\beta_{1}$ to vanish. For K=2 a modest imaginary multiplier survives, $\max_{k}\left|\beta_{k}\right|=0.346$, because the quadratic real part suppresses the density wherever the phase would otherwise leave [−π/2,π/2]; the mode moves from 3.52 to 3.25 against a sample mode of 2.66, a partial correction. For K=3 the search returns $\max_{k}\left|\beta_{k}\right|=9.8\times 10^{-4}$ and the two solutions again nearly coincide. The mode-correction advantage of the complex framework on a continuous, effectively unbounded support is therefore confined to intermediate constraint order.

**Figure A2:**
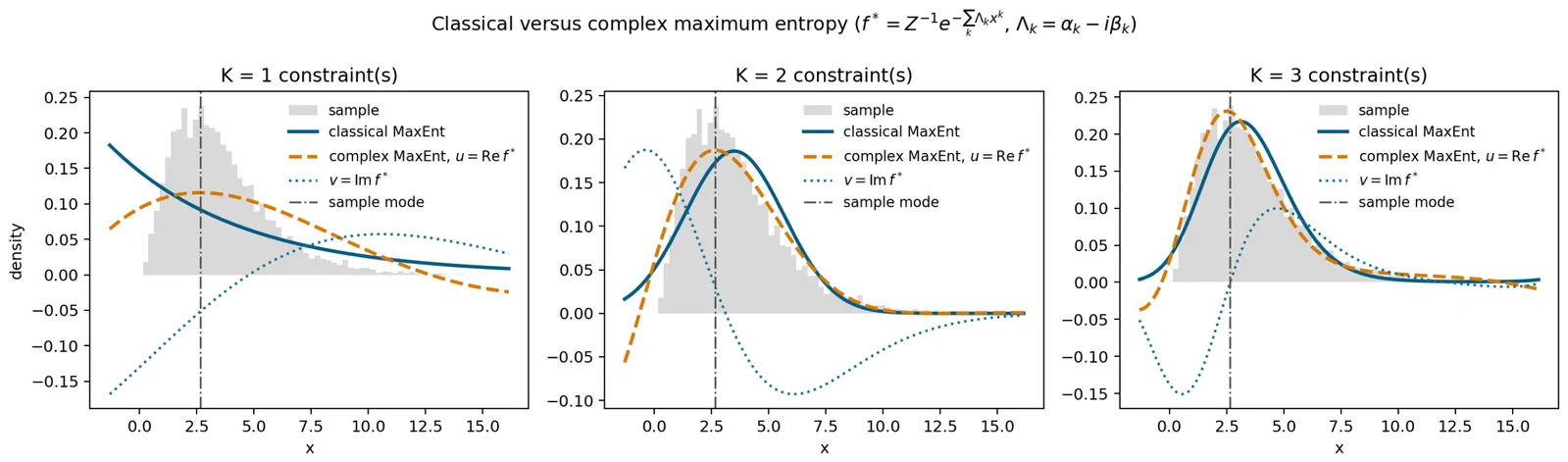
As Figure A1 with a non-zero admissibility tolerance.

## Data Availability

The Python script maxent_complex.py implementing the numerical examples of Appendix A is provided. No new data were created in this study.

## Abbreviations

The following abbreviations are used in this manuscript:

MaxEnt	Maximum entropy
CPDF	Complex probability density function
PDF	Probability density function
KDE	Kernel density estimation
QM	Quantum Mechanics

## Appendix A. Numerical Implementation: Python Code

### Appendix A.1. The Continuous Setting and the Example

The purpose of this appendix is to show the extension of the framework to a continuous support and to illustrate the mode-correction advantage of complex MaxEnt on synthetic data. The numerical implementation is provided in the accompanying script maxent_complex.py.

The worked example uses a synthetic sample of n=5000 observations from a Gamma distribution with shape a=3 and scale s=1.2. This distribution has mean μ=as=3.6, mode $x^{*}=\left(a-1\right)s=2.4$, and variance $\sigma^{2}=as^{2}=4.32$. It is right-skewed and has positive support, properties that make it representative of a broad class of physical and environmental data. Indeed, it is a classical illustration of modeling drop size distributions in the microphysics of precipitation for weather and climate models [24].

The classical MaxEnt with K=1 constraint, the prescribed mean μ, is the exponential distribution, whose mode is at the left boundary of the support rather than at the interior mode of the generating distribution. The example demonstrates that the complex extension, with the same single constraint on the real mean, can recover the interior mode by using the imaginary Lagrange multipliers as free parameters, rather than prescribing them from external knowledge.

### Appendix A.2. The Mode-Relaxation Formulation

The standard complex MaxEnt problem requires the imaginary moments $c_{k}^{\left(v\right)}=\int g_{k}\left(x\right)\;v^{*}\left(x\right)\;dx$ to be prescribed as inputs alongside the classical moments $c_{k}^{\left(u\right)}=\int g_{k}\left(x\right)\;u^{*}\left(x\right)\;dx$. As established in Section 1, the imaginary moments cannot be estimated from a real sample and must come from prior structural knowledge. When such knowledge is not available but a target mode location is known or estimated, for instance from the sample mode via kernel density estimation, the imaginary multipliers $\beta =(\beta_{1},\ldots ,\beta_{K})$ can instead be determined by solving the mode-relaxation problem: find β such that the observable distribution u∗(x)=Ref∗(x) has its maximum at a prescribed location $x^{†}$, while the real moments remain fixed at their estimated values ${\hat{c}}_{k}^{\left(u\right)}$.

Concretely, with $\alpha_{k}$ determined by the real moment conditions at each candidate β via an inner fixed-point solve, the mode-relaxation objective is

(A1) $$L\left(\beta \right)={\left(\frac{{\hat{x}}^{*}\left(\beta \right)-x^{†}}{\mathrm{span}}\right)}^{2}+\lambda_{1}\;{\left[\max(0,\;\eta (\beta ))-\varepsilon \right]}^{1/2}+\lambda_{2}\;{∥\beta ∥}^{2},$$

where ${\hat{x}}^{*}\left(\beta \right)=\arg\max_{x}u^{*}\left(x\right)$ is the mode of the observable density at the current β, η(β) is the integrated negative part of $u^{*}$, that is the (CP1) defect, ε is a user-supplied tolerance, and $\lambda_{1}$, $\lambda_{2}$ are regularization weights. The first term drives the mode toward the target; the second penalizes (CP1) violations, whose square-root growth makes the boundary soft, allowing a deliberate partial relaxation of the admissibility condition when the target mode is not achievable under strict (CP1); and the third is a Tikhonov term that prevents the phase multipliers from growing without bound on large supports. Setting ε=0 enforces strict admissibility throughout the search.

The role of ε should be stated explicitly, because it governs whether (CP1) is imposed as an axiom or as a soft penalty. For ε>0 the search accepts candidate densities whose observable part is slightly negative, the integrated negative part being bounded by ε; for ε=0 any candidate with $\min_{x}u^{*}\left(x\right)<0$ is rejected outright. All results reported below and in Figure A1 use ε=0, and the integrated negative part is quoted in each panel.

### Appendix A.3. Why Complex MaxEnt Improves on the Classical Solution

Figure A1 illustrates the improvement for K=1,2,3 power-moment constraints $g_{k}\left(x\right)=x^{k}$.

For K=1, the classical solution is the exponential distribution $p^{*}\left(x\right)\propto e^{-\lambda_{1}x}$, which is monotonically decreasing on the positive real line and places its mode at the left boundary of the support, far from the sample mode at $x^{†}\approx 2.4$. One might expect the imaginary multiplier to displace that mode into the interior, as it does on the bounded support of Section 4. Under strict enforcement of (CP1) it does not occur. With a single linear constraint the phase is $\beta_{1}x-\phi_{Z}$, which is unbounded whenever the support is unbounded, so the phase necessarily leaves [−π/2,π/2] and the admissibility condition forces $\beta_{1}=0$. The numerical search recovers exactly that, returning $|\beta_{1}|=6.9\times 10^{-5}$ and a solution indistinguishable from the classical one. With a non-zero admissibility tolerance, the solutions differ (Figure A2).

This is a specific instance of a general restriction, and it is worth stating because it delimits the applicability of the framework on continuous supports. Whenever the support is unbounded and the constraint functions are polynomials, the imaginary part of the Gibbs exponent is an unbounded polynomial and (CP1) forces every imaginary multiplier to vanish. Genuine freedom therefore exists on bounded or discrete supports, which is the setting of the die problem of Section 4, and only approximately on continuous supports, where it relies on the density being negligible outside a bounded region. That is why the effect reappears at K=2, where the quadratic term supplies the necessary decay.

For K=2, both the classical and complex solutions can produce interior modes, because the Gaussian-like classical distribution already has a free parameter, the variance, that locates its peak. The classical MaxEnt places the mode at a position determined jointly by the prescribed mean and variance; the complex MaxEnt, with an additional imaginary multiplier, can align the mode more precisely with the sample mode while simultaneously matching both classical moments.

This is the one case in which the effect survives strict enforcement of (CP3): the quadratic real part decays fast enough that the density is negligible wherever the phase would otherwise leave [−π/2,π/2], so an imaginary multiplier of 0.346 is admissible and the mode moves from 3.52 to 3.25 against a sample mode of 2.66. The advantage is reduced but still visible in Figure A1.

For K=3, the classical solution is flexible enough to produce a near-perfect fit to the sample histogram with three moment constraints, and the complex solution is close to it. The remaining difference is in the imaginary part v∗=Imf∗, which oscillates around zero and encodes the sub-population structure of the imaginary constraint, rather than contributing substantially to the mode location. This convergence shows that, as *K* grows, the classical and complex families approach the same distribution from different directions: classical MaxEnt gains shape flexibility through additional real constraints, while complex MaxEnt achieves a similar effect at smaller *K* by using the imaginary multipliers as shape parameters.

The key advantage of the complex approach is therefore most pronounced at *small K*: when the data are consistent with a small number of moment constraints but the classical exponential family cannot produce the correct mode, the complex extension provides that capability at no cost in terms of the classical constraints and without requiring additional moment measurements.

### Appendix A.4. Code Structure and Usage

The script maxent_complex.py implements the following workflow.

1. **Data ingestion.** A one-column text file is read via --data, or a synthetic Gamma sample is generated via --synthetic. The classical moments ${\hat{c}}_{k}^{\left(u\right)}=n^{-1}\sum_{i}x_{i}^{k}$ are estimated directly from the sample for k=1,2,3;
2. **Grid construction.** A bounded support is built by make_grid, extending the data range by a user-controlled fraction on each side. Boundedness of the support guarantees existence of the Gibbs solution for K=1 (exponential) and K=3, since a bounded Gibbs distribution always exists for odd-order polynomial exponents. For even *K* alone, the support must be explicitly bounded;
3. **Classical MaxEnt.** classical_maxent minimises the convex dual $\log Z\left(\lambda \right)+\lambda \cdot {\hat{c}}^{\left(u\right)}$ via BFGS with analytic gradient, to tolerance $10^{-12}$ on the moment residual;
4. **Complex MaxEnt.** When imaginary moments are prescribed via --imag, complex_maxent solves the stacked real and imaginary moment residuals
  (A2) Re∫gkf∗dx=c^k(u),Im∫gkf∗dx=ck(v),
  via the MINPACK hybr (modified Powell) root finder, to tolerance $10^{-13}$. The complex entropy Re(H[f∗])=−∫(ulog|f∗|−vθ)dx is computed from the solution and compared with the classical entropy;
5. **Mode-relaxation.** When --fitmode is specified, the classical solution is used to warm-start the inner α-solve, and the outer search over β proceeds in three stages: a coarse axis scan along each $\beta_{k}$ independently, a randomised perturbation phase, and a Nelder–Mead polish. The resulting β determines the imaginary constraints via ck(v)=Im∫gkf∗dx, which are then passed to complex_maxent for the final solution at full precision;
6. **(CP1) enforcement.** When --enforce-cp1 is specified, solve_admissible bisects the scale s∈(0,1] on the imaginary moments until $\int \min(u^{*},0)\;dx<\varepsilon$, reporting the largest admissible fraction. This identifies the achievability boundary for the prescribed imaginary moments on the given support, analogous to the discrete achievability table of Section 4.5.

Invocation examples:

python maxent_complex.py--data sample.txt--imag 0.15 0.0 0.0
python maxent_complex.py--synthetic--fitmode
python maxent_complex.py--data sample.txt--fitmode--cp1-tol 1e-4
python maxent_complex.py--data sample.txt--imag 0.5 0.0 0.0--enforce-cp1

The first call prescribes the imaginary moments directly; the second runs the mode relaxation on synthetic Gamma data and produces Figure A1; the third relaxes the (CP1) tolerance during the search; the fourth reports the achievability boundary for prescribed imaginary moments. All runs write a structured text report to maxent_results.txt, containing moments, multipliers, entropy, (CP1) diagnostics, and a tabulated distribution on the grid, together with a three-panel figure in maxent_pdfs.png.

### Appendix A.5. Key Functions

Listing A1 gives the two core numerical functions. All integrals are evaluated by the trapezoidal rule on the grid, which is adequate for smooth densities on a bounded support; finer quadrature could be substituted without changing the interface.

Listing A2 gives the mode-relaxation routine fit_mode, which implements the three-stage search described above. The inner function evaluate calls solve_alpha_given_beta, not shown, which solves the real moment equations for α at fixed β via a separate root find, and computes the objective (A1).

**Listing A1:** Core functions of maxent_complex.py: complex_gibbs evaluates the complex Gibbs distribution at given multipliers and complex_maxent solves the full moment system, returning the observable density, imaginary part, complex entropy, and (CP1) diagnostics. Integrals are computed by the trapezoidal rule on a uniform grid.

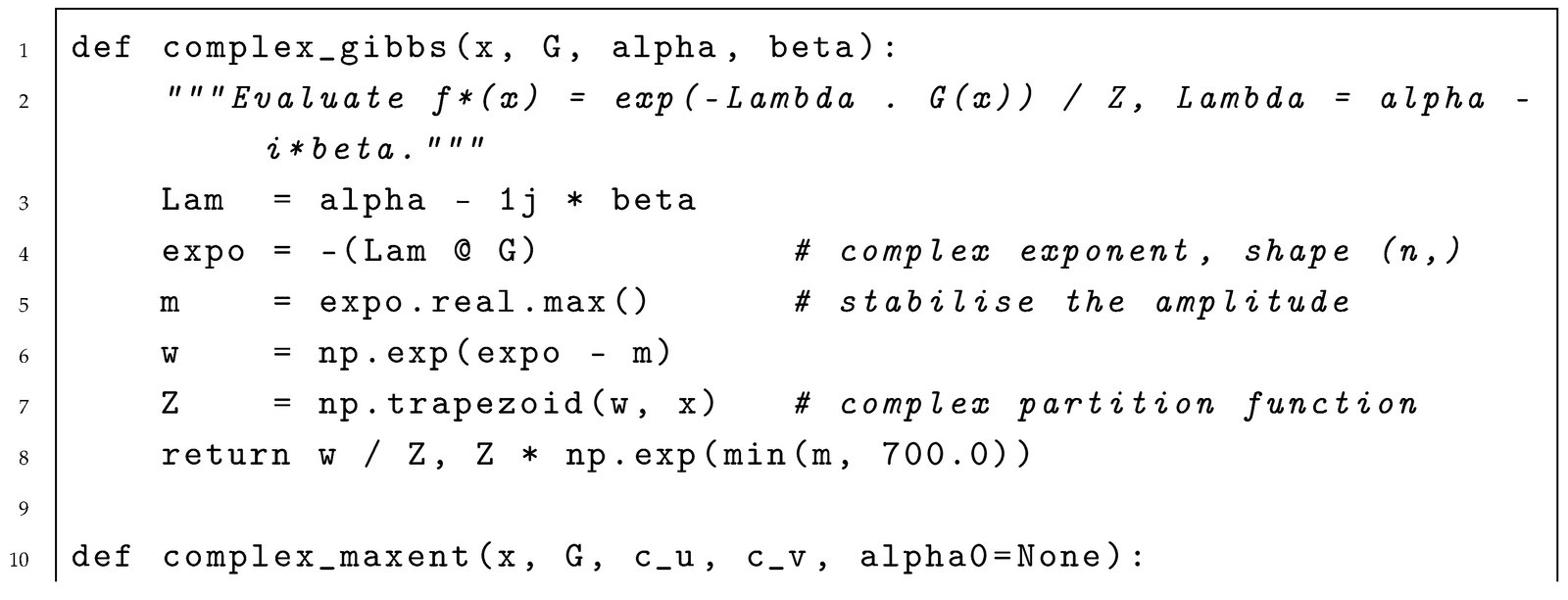

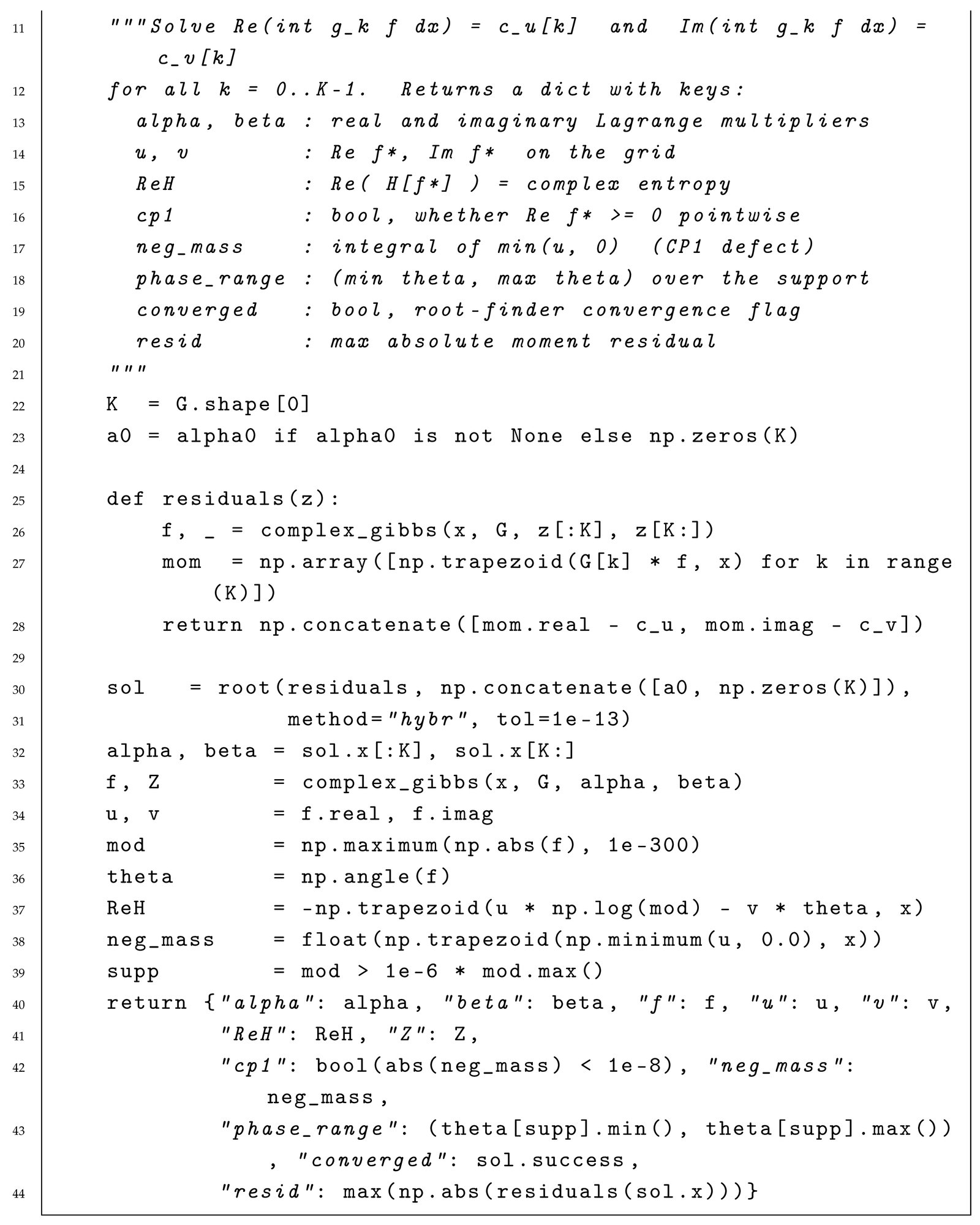

**Listing A2:** Mode-relaxation routine fit_mode. The function searches over the imaginary multipliers so that the mode of the observable density coincides with a prescribed target, while the real moments remain fixed. The three-stage search, axis scan followed by random perturbation and a Nelder–Mead polish, handles the discontinuity of the argmax and the plateau around zero phase. The cp1_tol parameter controls strictness of the admissibility condition during the search.
